# Galectin-3在非小细胞肺癌患者血清中的表达及其临床意义

**DOI:** 10.3779/j.issn.1009-3419.2020.102.03

**Published:** 2020-05-20

**Authors:** 德林 齐, 毅 张, 洪利 李, 若天 王, 坤 钱

**Affiliations:** 1 100053 北京, 首都医科大学宣武医院心脏外科 Department of Cardiac Surgery, Xuanwu Hospital, Capital Medical University, Beijing 100053, China; 2 100053 北京, 首都医科大学宣武医院胸外科 Department of Thoracic Surgery, Xuanwu Hospital, Capital Medical University, Beijing 100053, China

**Keywords:** 半乳糖凝集素3, 肺肿瘤, 肿瘤转移, Galectin-3, Lung neoplasms, Cancer metastasis

## Abstract

**背景与目的:**

肺癌是对人类健康威胁最大的疾病之一, 发病率高, 死亡率高。早期肺癌可以通过外科手术得到根治, 因此, 肺癌的早期发现及早期治疗尤其重要。血清肿瘤标志物是发现及诊断肺癌的一个重要手段。目前已知, 半乳糖凝集素3(Galectin-3)在多种恶性肿瘤中均有表达。该研究旨在探讨Galectin-3在非小细胞肺癌患者血清中的表达水平及其临床意义。

**方法:**

采用酶联免疫吸附法检测并比较69例非小细胞肺癌患者与77例健康人群外周血血清Galectin-3浓度, 并分析其表达水平与肺癌临床特征之间的关系。

**结果:**

Galectin-3在非小细胞肺癌患者外周血血清中的表达水平高于健康人群, 差异具有显著统计学意义(*P* < 0.01)；有淋巴结转移的非小细胞肺癌患者, Galectin-3表达水平显著高于无淋巴结转移患者, 差异有显著统计学意义(*P* < 0.01)；N2淋巴结转移的患者Galectin-3表达水平显著高于N1淋巴结转移的患者, 差异有显著统计学意义(*P* < 0.01)；临床Ⅲ期+Ⅳ期患者Galectin-3表达水平显著高于临床Ⅰ期+Ⅱ期患者, 差异有统计学意义(*P* < 0.05)。

**结论:**

非小细胞肺癌患者血清Galectin-3水平呈现高表达, 与肺癌转移相关, 可能是一种潜在的肺癌肿瘤标志物。

## Abstract (en)

**Background and objective:**

Lung cancer is one of the most dangerous diseases to human health, with high morbidity and mortality. It can be cured by surgery at early stage, therefore, the early detection and early treatment of lung cancer are especially important. Serum tumor markers play an important role in the detection and diagnosis of lung cancer. Galectin-3 is known to be expressed in a variety of malignant tumors. This study was to explore the serum levels of Galectin-3 and its clinical significance in non-small cell lung cancer (NSCLC) patients.

**Methods:**

The serum levels of Galectin-3 in peripheral blood were detected by enzyme linked immunosorbent assay (ELISA) in 69 NSCLC patients and 77 cases of healthy control subjects, and compared between the two groups. Then we analyze the correlations between the serum levels of Galectin-3 and the clinical features of lung cancer.

**Results:**

The serum levels of Galectin-3 in NSCLC patients were significantly higher than those of healthy control subjects (*P* < 0.01). The serum levels of Galectin-3 with lymph node metastasis were significantly higher than those of patients without lymph node metastasis (*P* < 0.01), and N2 lymph node metastasis had higher levels of serum Galectin-3 than those of N1 lymph node metastasis (*P* < 0.01). Clinical stage Ⅲ and stage Ⅳ patients had higher levels of serum Galectin-3 than those of clinical stage Ⅰ and clinical stage Ⅱ (*P* < 0.05).

**Conclusion:**

Our study showed the serum levels of Galectin-3 are highly expressed in NSCLC patients and are significantly related to lymph node metastasis. It may be a potential tumor marker for lung cancer.

肺癌是全球范围内癌症导致死亡的首要原因, 发病率高, 死亡率高, 但早期肺癌经根治性手术切除, 5年生存率可高达80%以上, 所以研究肺癌的生物学特点, 寻找早期发现肺癌的方法对于提高肺癌治愈率, 改善肺癌患者预后显得尤为重要。半乳糖凝集素3(Galectin-3)是凝集素家族(Galectins)中的一员, 最早发现于上皮组织。迄今为止, 共发现15种半乳糖凝集素, 分别命名为Galectin 1-15^[1]^。大量的研究认为Galectin-3在机体许多重要生理及病理过程中发挥作用, 如细胞生长及分化、上皮组织的修复、血管形成、炎症反应及肿瘤的发生进展等, 这可能与Galectin-3的在血浆及细胞核内的分布相关。本实验通过酶联免疫吸附法对Galectin-3在非小细胞肺癌(non-small cell lung cancer, NSCLC)患者血清中的表达水平进行了检验, 并对其临床意义进行了探讨。

## 1 资料与方法

### 1.1 一般资料

该研究共检测69例NSCLC患者外周血血清标本, 用于检测血清Galectin-3的肺癌患者外周血均取自首都医科大学宣武医院2013年2月-2013年11月胸外科收治的患者, 其中男性48例, 女性21例, 中位年龄65岁(43岁-84岁), 既往有吸烟史40例, 无吸烟史29例; 肿瘤最大径≤3 cm 22例, > 3 cm 47例; 鳞癌26例, 腺癌43例, 低分化19例, 中-低分化2例, 中分化12例, 高分化4例, 分化程度不详32例, 有淋巴结转移25例, 无淋巴结转移44例。临床研究开展前均与患者签署知情同意书, 并且经过院伦理委员会通过。

入组标准：①入院前未接受任何肺癌的特异性治疗, 包括手术、放射治疗、化疗、靶向药物治疗及生物治疗; ②经纤维支气管镜活检, 经皮肺针吸活检或术后病理证实为肺癌患者。根据世界卫生组织(World Health Organization, WHO)2015年颁布的肺癌组织学分型标准对肺癌组织进行分型, 并根据国际抗癌联盟(Union for International Cancer Control, UICC)2017年颁布的第8版肺癌肿瘤原发灶-淋巴结-转移(tumor-node-metastssis, TNM)分期系统进行肺癌分期。

健康对照组血清均取自我院体检中心同期健康体检者, 共77例, 男性56例, 女性21例, 中位年龄61岁(37岁-86岁)。

病例组与健康对照组之间性别及年龄无统计学差异(*P* > 0.05)。

### 1.2 研究方法

用EDTA抗凝管采肺癌患者及健康对照人群空腹外周血, 置4 ℃冰箱暂存, 24 h内送实验室, 经低温高速离心(2, 000 rpm, 4 ℃, 10 min)分离, 取血清分装后保存-80 ℃冰箱待检。采用双抗体夹心酶联免疫吸附法检测Galectin-3。酶联免疫吸附测定(enzyme linked immunosorbent assay, ELSIA)试剂盒购自美国R & D公司, 严格按照试剂盒说明书操作。

研究通过标准Galectin-3蛋白浓度的溶液及其OD_450_绘制标准曲线, 并分别检测健康人群与肺癌患者血清的OD_450_, 以此计算其血清Galectin-3浓度, 并对健康人群与肺癌患者及早期肺癌人群的血清Galectin-3水平分别进行比较; 在非小细胞肺癌患者中, 根据患者年龄、性别、吸烟史、肿瘤大小、病理类型、肿瘤分化程度、淋巴结转移情况及临床分期进行比较。

### 1.3 统计学方法

对两组数据采用SPSS 17.0统计软件(SPSS INC, IL, USA)进行统计学分析。由于所测肺癌患者血清Galectin-3浓度均为计量资料, 所有数据进行正态性检验, 不符合正态分布的数据进行非参数检验(*Mann-Whitney U* test), 符合正态分布并具有方差齐性的数据进行*t*检验; 全部显著性检验均为双侧检验, 当*P* < 0.05时被认为差异有统计学意义, *P* < 0.01表示差异具有显著统计学意义。

## 2 结果

### 2.1 外周血血清Galectin-3浓度比较

NSCLC患者外周血血清Galectin-3浓度显著高于健康人群[(1.38±0.75) ng/mL *vs* (0.92±0.53) ng/mL], 两者之间差异有显著统计学意义(*P*=0.002)。早期肺癌患者(Ⅰ期+Ⅱ期)外周血血清Galectin-3浓度与健康人群比较[(1.09±0.82) ng/mL *vs* (0.92±0.53) ng/mL], 差异无统计学意义(*P* > 0.05)。NSCLC患者外周血血清Galectin-3浓度与患者年龄、性别、吸烟史、肿瘤大小、组织学分型及分化程度无相关性。见表 1。

**1:** 肺癌患者血清Galectin-3浓度与临床特征的关系(Mean±SD, ng/mL) Correlation between serum levels of Galectin-3 and clinical features (Mean±SD, ng/mL)

Characteristic	*n*	Galectin-3 level	*P*
Age (yr)			0.062
≤60	20	1.40±0.64	
> 60	49	1.19±0.85	
Gender			0.965
Male	48	1.26±0.82	
Female	21	1.31±0.90	
Smoking history			0.672
Yes	40	1.28±0.79	
No	29	1.16±0.63	
Tumor size			0.825
≤3 cm	22	1.32±0.97	
> 3 cm	47	1.21±0.74	
Histology			0.652
Squamous carcinoma	26	1.18±0.80	
Adenocarcinoma	43	1.29±0.85	
Pathological grade			0.185
Well and moderately differentiated	16	1.38±0.80	
Poorly differentiated	21	1.06±0.64	
Lymph node metastasis			0.005
Yes	25	1.63±0.87	
No	44	1.03±0.72	
Lymph node metastasis			0.007
N1	12	0.88±0.38	
N2	8	1.76±0.89	
Clinical stage			0.044
Ⅰ+Ⅱ	40	1.09±0.82	
Ⅲ+Ⅳ	29	1.50±0.82	

### 2.2 不同临床特征间Galectin-3浓度的比较

① 有淋巴结转移的NSCLC患者血清Galectin-3浓度显著高于无淋巴结转移患者血清Galectin-3浓度。②同侧纵隔内及(或)隆突下淋巴结(N2)转移患者与同侧支气管周围及(或)同侧肺门淋巴结以及肺内淋巴结(N1)转移患者比较, 血清Galectin-3浓度显著升高。③晚期肺癌患者(Ⅲ期+Ⅳ期)血清Galectin-3浓度显著高于早期肺癌患者(Ⅰ期+Ⅱ期)血清Galectin-3浓度。见表 1。

## 3 讨论

现已证实, Galectin-3在甲状腺癌、乳腺癌、结肠癌等多种肿瘤组织中均有表达, 且与肿瘤的恶性度具有正相关性, 即, 对肿瘤的形成、进展、转移及复发等生物学行为具有促进作用。目前研究发现, Galectin-3对肿瘤的促进作用可能主要涉及以下几种机制：①Galectin-3的抗凋亡活性有研究^[2]^发现, 敲除口腔鳞状上皮细胞的*Galectin-3*基因可以诱导细胞凋亡, 进一步的研究^[3]^提示, 这可能是通过影响凋亡调节因子的表达实现的。②促进细胞生长及增殖细胞生长及增值是肿瘤局部进展的基础, Prado等^[4]^发现, 抑制Galectin-3的表达能抑制结肠癌细胞增殖。③促进新生血管形成新生血管形成是肿瘤形成及远处转移的必要条件, Galectin-3促进血管形成的作用机制可能与肿瘤相关巨噬细胞(如M2型巨噬细胞)的渗出有关^[5]^。④促进细胞黏附。Colomb等^[6]^的研究发现, Galectin-3可与细胞表面的糖蛋白CD146结合, 进而诱导内皮细胞分泌肿瘤转移促进因子。

关于Galectin-3在血清中的研究并不鲜见, 目前已有研究^[7]^发现, 乳腺癌患者血清Galectin-3水平较健康人群增高, 更有研究^[8]^发现, 血清Galectin-3水平对于胰腺及胆道系统肿瘤具有诊断意义。

目前, 对于Galectin-3与肺癌的相关性研究主要集中在肺癌组织中, 其在肺癌血清中的研究相对少见, 研究结果多提示Galectin-3与肺癌的恶性度呈现正相关性, 这与本研究的发现是一致的。Galectin-3对肿瘤的促进作用机制, 可能与Galectin-3促进肺肿瘤细胞对微血管的侵入及促进新生血管的形成有关, 该作用可能与缺氧环境下, Galectin-3影响细胞因子RHoA在细胞膜的定植^[9]^及Galectin-3抑制免疫渗出^[10]^, 尤其是巨噬细胞的渗出相关。

本研究发现, 肺癌患者血清Galectin-3表达水平显著高于健康人群, 这提示, Galectin-3有望成为一种新的肿瘤标志物; 而早期肺癌患者血清Galectin-3水平与健康人群比较无明显差异, 在肺癌患者, 有淋巴结转移患者血清Galectin-3水平显著高于无淋巴结转移患者, 临床Ⅲ期+Ⅳ期肺癌患者血清Galectin-3水平显著高于临床Ⅰ期+Ⅱ期患者, 提示Galectin-3可能淋巴结转移相关, 并与肺癌的恶性度具有正相关性, 可能对于肺癌的预后判断具有一定的价值。该研究还发现, N2淋巴结转移的患者血清Galectin-3水平显著高于N1淋巴结转移患者, 虽与Galectin-3对肿瘤的正性促进作用相吻合, 但由于样本量较小, 故其价值仍值得商榷。该研究所示, 早期肺癌患者血清Galectin-3水平与健康人群相比, 并无显著差异, 这可能提示, 作为肿瘤标记物, 血清Galectin-3的敏感性较差; 而鳞癌与腺癌患者比较, 血清Galectin-3水平无差异, 则可能体现出该标记物的组织特异性比较差。由于实验条件限制, 该研究样本量并不足够大, 并且没有对患者的预后进行追踪跟访, 缺乏血清Galectin-3与预后相关性的研究, 这是该研究的一个不足之处。因肿瘤的形成、进展、转移及复发是一个非常复杂的过程, 前述Galectin-3对肿瘤的促进作用的机制, 可能是一系列复杂机制的不同环节, Galectin-3对肿瘤的作用的具体机制有待进一步深入认识。

随着科技的不断进步, 现代医学对于肿瘤的形成及进展的认识逐渐深入至分子生物学层面, 希望该研究能够为肺癌的诊断、治疗及预后判断提供新的思路和方法, 并能够为进一步探讨肺癌的形成及进展机理提供一定的依据。
